# Hepatoprotective and antifibrotic effects of *trans*-chalcone against bile duct ligation-induced liver fibrosis in rats

**DOI:** 10.22038/IJBMS.2023.68342.15040

**Published:** 2023

**Authors:** Faezeh Javadi, Mahsa Ale-Ebrahim, Parvaneh Mohseni-Moghaddam, Pejman Mortazavi, Zahra Mousavi, Ahmad Asghari

**Affiliations:** 1 Department of Pharmacology and Toxicology, Faculty of Pharmacy and Pharmaceutical Sciences, Tehran Medical Sciences, Islamic Azad University, Tehran, Iran; 2 Department of Physiology, Faculty of Medicine, Tehran Medical Sciences, Islamic Azad University, Tehran, Iran; 3 Department of Pathology, Faculty of Specialized Veterinary, Science and Research Branch, Islamic Azad University, Tehran, Iran

**Keywords:** Anti-oxidant, Bile duct ligation, Cholestasis, Hepatic fibrosis, Trans-chalcone

## Abstract

**Objective(s):**

Several lines of research have shown that hepatic fibrosis is one of the leading causes of death worldwide. *Trans*-chalcone is a flavonoid precursor with anti-oxidant and anti-inflammatory effects. The present study was conducted to examine the antifibrotic properties of *trans*-chalcone on bile duct ligation (BDL)-induced liver cholestasis in rats.

**Materials and Methods:**

Following the BDL operation, *trans*-chalcone at doses of 12, 24, and 50 mg/kg was administered orally once a day for 45 consecutive days. Serum levels of liver indices, including alkaline phosphatase (ALP), alanine aminotransferase (ALT), aspartate aminotransferase (AST), total and direct bilirubin, and lipid profile in addition to blood urea nitrogen (BUN) and creatinine, were measured. Additionally, catalase (CAT) and superoxide dismutase (SOD) activities were assessed in liver homogenates. Histopathological evaluations were performed using Masson trichrome (MT) and hematoxylin and eosin (H&E) staining.

**Results:**

The elevated levels of liver enzymes, total and direct bilirubin, BUN, creatinine, cholesterol, triglyceride, and low-density lipoprotein (LDL) induced by BDL were significantly reduced following *trans*-chalcone administration; while serum level of high-density lipoprotein (HDL) increased. Besides, treatment with *trans*-chalcone elevated the activities of CAT and SOD in the liver tissues of the animals with BDL surgery. According to MT and H&E staining, BDL-induced histopathological changes, including infiltration of inflammatory cells, hepatocyte necrosis, ductal hyperplasia, and collagen deposition were ameliorated using *trans*-chalcone administration.

**Conclusion:**

It can be concluded from the present study that *trans*-chalcone, possibly by its anti-oxidant and anti-inflammatory properties, may exert hepatoprotective and antifibrotic effects in BDL-induced liver fibrosis.

## Introduction

Increasing evidence has shown that the liver has a vital role in food and drug metabolism. Fatty liver, viral hepatitis, and cholestatic fibrosis are among the most common disorders of the liver (1). Liver fibrosis is caused by abnormal and high deposition of extracellular matrix (ECM), disrupting the regular liver architecture and also liver function (2). Aggregation of toxic bile acids in the cholestatic liver influences the oxidant-anti-oxidant status, promoting reactive oxygen species (ROS) production (3, 4). Damage to the hepatocytes can be caused by free radicals due to the alkylation of proteins, nucleic acids, and lipids, as well as lipid peroxidation. Oxidative stress leads to the synthesis of pro-inflammatory cytokines and promotes hepatic stellate cells (HSCs) activation (5, 6). Numerous studies suggest that anti-oxidant supplements significantly inhibit lipid peroxidation and liver fibrosis (7, 8).

There is an increasing demand for the prevention and treatment of cholestatic liver fibrosis. Current investigations have focused on finding new substances with few or no side effects compared to synthetic drugs.* Trans*-chalcone is a flavonoid precursor, exerting various pharmacological properties, such as antidiabetic, hepatoprotective, analgesic, anti-oxidant, and anti-inflammatory activities (8-13). The protective property of *trans*-chalcone against non-alcoholic steato-hepatitis (NASH) induced by a high-fat diet (HFD) is caused by improvement in liver lipid metabolism (10). Moreover, *trans*-chalcone administration ameliorated liver fibrosis in high-cholesterol diet (HCD)-fed mice by increasing the anti-oxidant enzymes and modulating the lipid profile (7). Another study has also shown that the cytoprotective activity of *trans*-chalcone in hepatocellular carcinoma cells is through reducing oxidative stress (14). Antifibrotic effects of *trans*-chalcone on the experimental model of liver injury induced by carbon tetrachloride (CCL4) and paracetamol have also been investigated previously (8). However, no study has examined whether *trans*-chalcone is efficient in the treatment of liver fibrosis induced by bile duct ligation (BDL). Thus, the current study aims to evaluate the hepatoprotective effects of *trans*-chalcone by assessing its anti-oxidant activity and also its beneficial effects on lipid profile and liver indices in the BDL model of liver fibrosis. 

## Materials and Methods

***Animals***

In this research, 35 male Wistar rats weighing 230–280 g were applied. The animals had free access to standard pellet and distilled water and were kept under 12 hr light-dark cycles. All experiments performed on animals were in accordance with the Guide for the Care and Use of Laboratory Animals (National Institutes of Health, USA). Moreover, Animal Ethics Committee approval has been obtained from the Faculty of Medical Sciences, Islamic Azad University, Tehran, Iran.

***BDL operation and experimental procedures***

Each male Wistar rat was randomly put in one of the following seven groups (each group had five animals): (1) control group: rats received 1 ml of sunflower oil, *trans*-chalcone solvent, once a day for 45 days; (2) control+50: animals were given *trans*-chalcone orally at the dose level of 50 mg/kg daily for 45 days; (3) sham group: animals with laparotomy surgery and without BDL. They also received 1 ml of sunflower oil daily for 45 days; (4) BDL group: animals with BDL surgery were administered sunflower oil; (5–7) BDL+12, BDL+24, and BDL+ 50 groups: rats with BDL surgery were given *trans*-chalcone orally at doses of 12, 24, and 50 mg/kg, respectively. Following dissolving of *trans*-chalcone in sunflower oil, 1 ml of the solution was administered orally once a day over a period of 45 days. *Trans*-chalcone administration was started from the day of BDL surgery. The *trans*-chalcone doses used in the current research were based on a previously published article by Karkhaneh and colleagues (7). The BDL surgery was accomplished according to a standard method (15). Concisely, anesthesia was induced in rats with intraperitoneal injections of ketamine (90 mg/kg) and xylazine (10 mg/kg). Afterward, an incision was made in the midline of the abdomen. Following identifying the common bile duct, it was closed in two regions, including below the hepatic duct junction and before the pancreatic duct entrance. Subsequently, the common bile duct, located between the two ligated points, was cut. Finally, sterile saline in a volume of 2 ml was added into the peritoneal cavity, and then the abdominal incision was sutured. Afterward, each animal was placed on a heating pad to recover (16). In sham-operated groups, an incision was made in the abdomen, but the common bile duct was not closed.

***Compounds***

*Trans*-chalcone was purchased from Sigma–Aldrich, St. Louis, MO, USA. To measure direct and total bilirubin, aspartate aminotransferase (AST), alkaline phosphatase (ALP), alanine aminotransferase (ALT), catalase (CAT), blood urea nitrogen (BUN), creatinine, total cholesterol, triglyceride, high-density lipoprotein (HDL), and low-density lipoprotein (LDL) commercial kits were obtained from Pars Azmoon Company, Iran. The assay kit for measuring the activity of superoxide dismutase (SOD) was procured from Dojindo Laboratories, Kumamoto, Japan.

***Sampling and biochemical evaluations***

When the animal experiments were completed, the rats were kept fasting for 18 hr. Each animal was anesthetized with ketamine (90 mg/kg) and xylazine (10 mg/kg), and then blood samples and the whole liver were taken from rats. Afterward, each rat was euthanized with an overdose of ketamine and xylazine. To assess histological alterations, a piece of liver tissue was promptly put in 10% formaldehyde for fixation. To measure CAT and SOD activities, another portion of the liver tissue was homogenized.

For serum preparation, the blood samples, taken from the animals, were first placed at room temperature for 30 min. Afterward, they were centrifuged for 10 min at 1000 × g at 37 °C. The serum levels of AST, ALT, and ALP, total and direct bilirubin, BUN, creatinine, LDL, HDL, triglyceride, and cholesterol were measured using commercial kits (17, 18). 

***Measuring the activities of CAT and SOD in liver tissue***

To prepare the liver homogenate, liver tissues were placed in phosphate buffer (50 mM, pH 7.0) and homogenized. Then, homogenized tissues were centrifuged for 30 min at 800 × g at 4 °C, and the supernatant was utilized to measure the activity levels of CAT and SOD enzymes. The level of CAT activity was measured using a spectrophotometric assay explained by Aebi (19). In brief, following the mixing of 0.2 ml of each sample with 1.2 ml of 50 mM phosphate buffer (pH 7.0), 1.0 ml of 30 mM H_2_O_2_ solution was added, and the reaction was begun. After that, the changes in absorbance were recorded at 240 nm at 30-second intervals for three minutes. CAT activity is indicated in unit/mg of protein.

Hepatic SOD activity was measured using a commercial kit (Dojindo Laboratories, Kumamoto, Japan). Briefly, 20 μL of samples were mixed with an assay reagent containing a water-soluble tetrazolium salt (WST-1). Afterward, the prepared mixtures were incubated at 37 °C for 20 min. Superoxide anions reduce WST-1 to WST-1 diformazan. The amount of WST-1 diformazan produced was measured at 450 nm. The dismutation of superoxide radicals is catalyzed by SOD and consequently inhibits the reduction of WST-1 (20).

***Histopathological examination***

MT and H&E staining was used to identify the histopathological alterations, including inflammation, fibrosis, necrosis, and bile-duct hyperplasia. According to the average of 10 random fields/slide, a single score was assigned to each sample (21). The extent of liver injury was scored as follows: necrosis: none= 0; focal necrosis in < 25% of the liver tissue= 1; focal necrosis in 25–50% of the liver tissue=2; extensive, but focal necrosis=3; global hepatocyte necrosis=4. Fibrosis: none=0; portal fibrosis=1; septal formation=2; marked bridging fibrosis=3; Cirrhosis=4. Hyperplasia in bile ducts: None= 0; hyperplasia in < 25% of each liver lobule= 1; hyperplasia in 25–50% of each liver lobule= 2; extensive but focal bile duct hyperplasia= 3; global ductal hyperplasia= 4. Inflammation: None= 0; focal inflammation in < 25% of the liver tissue= 1; focal inflammation in 25–50% of the liver tissue= 2; extensive but focal inflammation= 3; global inflammation= 4 (22).

***Statistical analyses***

Data from this study were presented as mean ± SEM. One-way ANOVA followed by a Tukey *post hoc* test was used to analyze the statistical differences between experimental groups. Data analyses were performed using the SPSS software (version 24).

When the *P*-value was less than 0.05, statistical differences were considered significant.

## Results

***Biochemical analysis***

**Figure 1:**
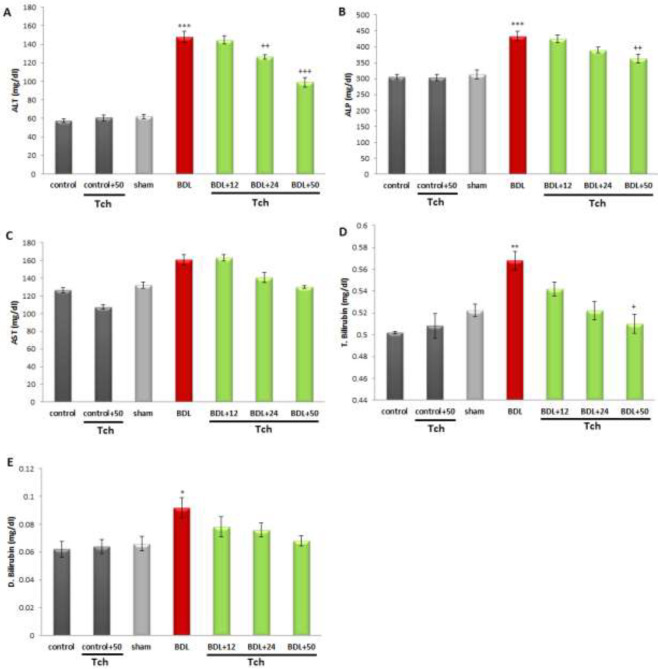
Comparing the serum levels of liver indices, including ALT (A), ALP (B), AST (C),total (D), and direct bilirubin (E), between rat experimental groups

A one-way ANOVA indicated that BDL markedly elevated the serum levels of liver injury biomarkers, including ALP and ALT, versus the sham group (*P*<0.001, for each) (Figure 1). Increased ALT level was significantly reduced in the BDL animals, which were administered *trans*-chalcone at doses of 24 and 50 mg/kg (*P*<0.01 and *P*<0.001, respectively). Additionally, *trans*-chalcone at the dose of 50 mg/kg could diminish the serum level of ALP (*P*<0.01) in the animals with BDL surgery. *Trans*-chalcone also decreased the serum AST level in the treatment groups to the level of the sham group. As shown in Figure 1, the serum total and direct bilirubin levels (*P*<0.01 and *P*<0.05, respectively) were significantly elevated in the animals with BDL surgery in comparison with the sham group. Treatment of the BDL animals with *trans*-chalcone at the dose of 50 mg/kg markedly diminished the serum level of total bilirubin (*P*<0.05), however, there was no remarkable difference in the serum level of direct bilirubin between the BDL group and treatment groups.

**Figure 2:**
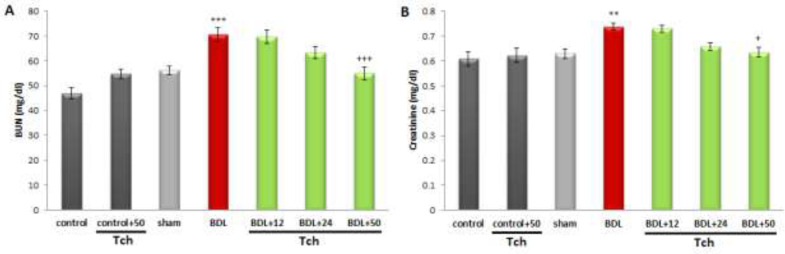
Comparing the serum levels of BUN (A) and creatinine (B) between rat experimental groups

**Figure 3:**
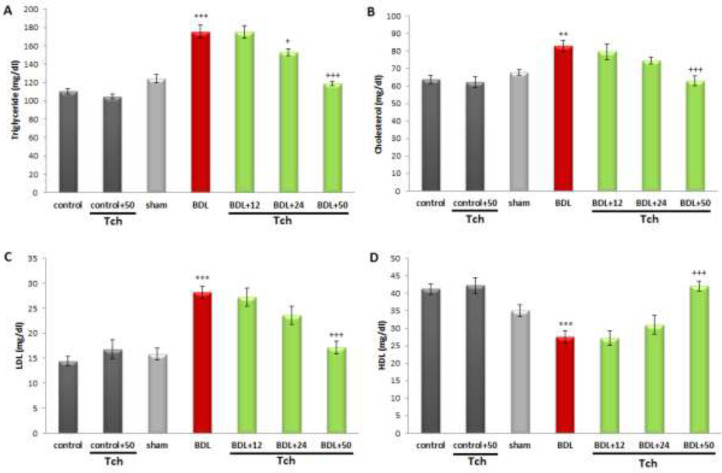
Comparison of serum levels of lipid profile, including triglyceride (A), cholesterol (B), LDL (C), and HDL (D) between rat experimental groups

Also, BDL markedly elevated the serum levels of BUN, creatinine (*P*<0.001 and *P*<0.01, respectively), cholesterol (*P*<0.01), triglyceride, and LDL (*P*<0.001, for each) compared to the sham group. On the other hand, the serum level of HDL was markedly decreased in the BDL group versus the sham group (*P*<0.001). As shown in Figures 2 and 3, treatment with *trans*-chalcone at the dose of 50 mg/kg significantly restored the serum levels of BUN, creatinine (*P*<0.001 and *P*<0.05, respectively), cholesterol, HDL, and LDL (*P*<0.001, for each) when compared with the BDL group. Moreover, treatment with *trans*-chalcone at dose levels of 24 and 50 mg/kg could remarkably attenuate the increased level of triglycerides induced by BDL (*P*<0.05 and *P*<0.001, respectively).

**Figure 4:**
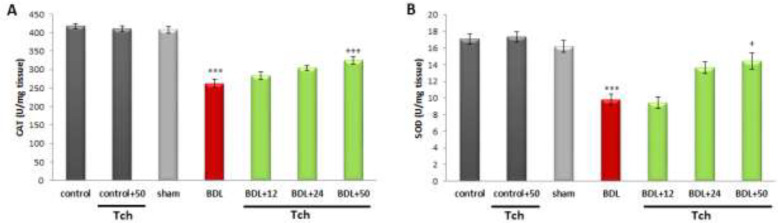
Comparison of the catalase and SOD activities in the liver tissue between rat experimental groups

Figures 4A and B show that CAT and SOD activities were significantly diminished in the liver of the BDL group versus the sham group (*P*<0.001, for each). In the animals with BDL surgery, *trans*-chalcone administration at the dose level of 50 mg/kg could significantly elevate SOD (*P*<0.05) and CAT (*P*<0.001) activities versus the BDL group. Besides, there were no significant differences in the levels of studied variables mentioned above between the control group, the control group that received *trans*-chalcone, and the sham group. 

As the results show, treatment with* trans*-chalcone in a dose-dependent manner has caused changes in the investigated parameters. In addition, the dose of 50 mg/kg was the best dose to decrease liver indices, modulate lipid profile, and elevate anti-oxidant activity.

***Trans-chalcone improved liver fibrosis***

**Figure 5:**
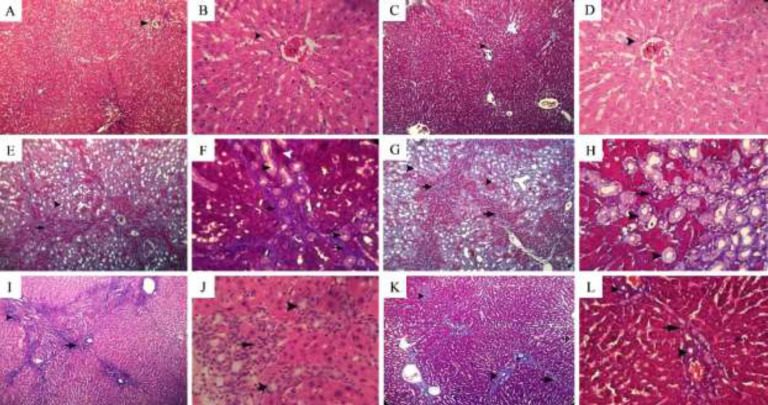
Evaluation of liver fibrosis in rat experimental groups using MT and H&E staining

Findings have shown that histological scores of liver injury in the BDL group were remarkably higher than those in the sham group, and treatment with *trans*-chalcone at doses of 24 and 50 mg/kg could markedly ameliorate histopathologic scores in the livers of BDL animals (Table 1). Histological results obtained from MT and H&E staining have shown that liver fibrosis indices, which include collagen deposition, infiltration of inflammatory cells (lymphocytes), hepatocyte necrosis, and ductal hyperplasia, were observed in the BDL animals. Treatment of the BDL animals with *trans*-chalcone at doses of 24 and 50 mg/kg could significantly reduce all the mentioned fibrosis indices. Additionally, in the liver tissues of the sham group and the control+ *trans*-chalcone, no histological abnormality was observed in comparison with the control group. Liver histology was normal with intact sinusoids, hepatocyte cells, and portal tract (Figure 5).

*Trans*-chalcone at the dose of 50 mg/kg could significantly reduce the serum levels of ALT, ALP, and total bilirubin in the animals with BDL surgery. *P<0.05, **P<0.01, and ****P<0.001 versus animals in the sham group, +*P< 0.05, ++*P<0.01, and +++*P<0.001 compared with animals in the BDL group (n= 5/group). ALT Alanine aminotransferase; ALP, Alkaline phosphatase; AST, Aspartate aminotransferase; BDL, Bile duct ligation; T. Bilirubin, Total bilirubin; D. Bilirubin, Direct bilirubin; Tch, *Trans*-chalcone

Trans-chalcone at the dose of 50 mg/kg could significantly reduce the mentioned parameters

**P<0.01 and ***P<0.001 versus animals in the sham group, +*P*<0. 05 and +++*P*<0.001 compared with animals in the BDL group (n= 5/group)

BDL, Bile duct ligation; BUN, Blood urea nitrogen; Tch, *Trans-*chalcone

In the BDL animals, trans-chalcone at the dose of 50 mg/kg could significantly ameliorate lipid profile

**P<0.01 and ***P<0.001 versus animals in the sham group, +*P*< 0.05 and+++*P*< 0.001 compared with animals in the BDL group (n= 5/group)

BDL, Bile duct ligation; LDL, Low-density lipoprotein; HDL, High-density lipoprotein; Tch, *Trans*-chalcone

In the animals with BDL surgery, *trans*-chalcone at the dose level of 50 mg/kg remarkably elevated the activity of the mentioned anti-oxidants

***P<0.001 versus animals in the sham group, +*P*<0.05 and +++*P*<0.001 compared with animals in the BDL group (n= 5/group)

BDL, Bile duct ligation; SOD, Superoxide dismutase; CAT, Catalase; Tch, *Trans*-chalcone

**Table 1:** Scores of liver injury

^a^Score of liver injury				
Groups	Inflammation	Ductal hyperplasia	Necrosis	Collagen deposition (fibrosis)
Control	0	0	0	0
Control+ *trans*-chalcone (50 mg/kg)	0	0	0	0
Sham	0	0	0	0
BDL	3***	4***	2***	3***
BDL+ *trans*-chalcone 12 mg/kg	2	4	2	2
24 mg/kg	1.18^++^	3.01^+^	1.96	2/01
50 mg/kg	0.98^+++^	1.24^+++^	1.17^++^	1.0^+++^

***P< 0.001 versus rats in the sham group, ++*P*< 0.01, and +++*P*< 0.001 versus rats in the BDL group

aInflammation: 0, none; 1, less than 25% of the liver tissue is involved with focal inflammation; 2, 25-50% of the liver tissue is involved with focal inflammation; 3, extensive but focal inflammation. Hyperplasia in bile ducts: 0, none; 1, less than 25% of each liver lobule is involved with hyperplasia; 2, 25–50% of each liver lobule is involved with hyperplasia; 3, extensive but focal hyperplasia. Necrosis: 0, none; 1, less than 25% of the liver tissue is involved with focal necrosis; + 2, 25–50% of the liver tissue is involved with focal necrosis; 3, extensive but focal necrosis. Fibrosis: 0, none; 1, portal fibrosis; 2, septal formation; 3, marked bridging fibrosis

(A) Control (100, MT staining), (B) Control (× 400, H&E staining), (C) control+ *trans*-chalcone at the dose of 50 mg/kg (×100, MT staining), (D) control+ *trans*-chalcone at the dose of 50 mg/kg (×400, H&E staining), (E and F) BDL (MT staining with×100 and ×400 magnification, respectively), (Gand H) BDL+ *trans*-chalcone at the dose of 12 mg/kg (MT staining with×100 and ×400 magnification, respectively), (I and J) BDL+ *trans*-chalcone at the dose of 24 mg/kg (H&E staining with×100 and ×400 magnification, respectively), and (K and L) BDL+ *trans*-chalcone at the dose of 50 mg/kg (MT staining with×100 and ×400 magnification, respectively). In control and control+ *trans*-chalcone groups (A-D), no histological abnormality was seen in the liver tissue, and CV and hepatocytes (arrowheads) around it were normal. Ductal hyperplasia (arrowheads) and extensive bridging fibrosis (arrow) were observed in the liver of the BDL group (E and F). Liver fibrosis was remarkably ameliorated by *trans*-chalcone treatment (G-L)

BDL, Bile duct ligation; CV, Central vein; H&E, Hematoxylin and eosin; MT, Masson trichrome

## Discussion

The current study evaluated hepatoprotective and anti-ﬁbrotic properties of *trans*-chalcone in BDL-induced liver ﬁbrosis. To indirectly evaluate liver status, the serum levels of ALT, ALP, and AST were assessed. BDL caused a marked increase in the serum levels of ALT and ALP. Several mechanisms have been proposed to explain the cytotoxic effects of cholestasis-mediated bile acid accumulation on liver tissue. Bile acids promote cell membrane disruption by their detergent effects on lipid molecules (23), and also by stimulating ROS production, leading to some modifications in nucleic acids, proteins, and lipids, eventually causing damage to hepatocytes (24). Besides, accumulation of bile acids results in Kupffer cell-mediated ROS generation, which in turn increases hepatocyte damage (23). Accordingly, hepatocytes, which contain high levels of ALP, ALT, and AST, release these enzymes into the bloodstream (25, 26). Treatment with *trans*-chalcone markedly lowered the serum levels of these liver enzymes in animals with BDL surgery, and this effect of *trans*-chalcone has also been demonstrated in other animal models (7, 8, 10, 27-29). It has been revealed that *trans*-chalcone, by inhibiting hepatic inflammation, can decrease serum liver enzyme levels (27). Karkhaneh and colleagues have also reported that *trans*-chalcone by increasing anti-oxidant defense, reduces liver injury and therefore decreases serum levels of liver enzymes in HCD-fed mice (7). In a study conducted by Karimi-Sales *et al*., it was indicated that *trans*-chalcone by alteration of the hepatic levels of several microRNAs could reduce serum levels of liver enzymes and hepatic inflammation and consequently inhibit the transition from steatosis to NASH(29).

Total bilirubin and direct bilirubin are two other parameters whose serum levels were augmented after BDL surgery in this study. The major indicator of cholestasis is elevation in the serum level of total conjugated bilirubin. Following cholestasis, conjugated bilirubin excretion into the bile is decreased, and it would efflux back into the bloodstream. It seems that due to weakened tight junctions between hepatocyte cells, bilirubin regurgitates into the blood. In addition, decreased rate of conjugation caused by hepatocellular injury in BDL animals increases serum direct bilirubin levels (30). In animals with BDL, oral administration of *trans*-chalcone could reduce the serum levels of total and direct bilirubin, which is in line with previous studies (7, 8). The reduction in the serum levels of direct and total bilirubin by *trans*-chalcone in HCD-fed animals has been attributed to its anti-oxidant activity (7).

The BDL model of liver fibrosis is reported to be associated with decreased SOD and CAT activities in the liver tissue (31). In the current study, CAT and SOD activities remarkably declined in liver tissue following BDL operation, and co-treatment with *trans*-chalcone elevated their activities at the dose level of 50 mg/kg. It is well-known that detoxification of free radicals is performed by SOD and CAT, and these anti-oxidants are also reported to reduce oxidative stress in cholestatic liver fibrosis (31). *In vitro* assessments have shown that hepatocytes are protected against toxic bile salts by the anti-oxidant activity of CAT and SOD (32). Anti-oxidant activity of *trans*-chalcone has also been previously reported, which is in agreement with the current study (7, 8, 14, 33, 34)

Cholestasis is accompanied by hyperlipidemia and remarkable changes in the lipid profile. In response to cholestasis and bile duct obstruction, a decrease in serum HDL level occurs, while the serum levels of LDL and total cholesterol increase (35, 36). Defects in the clearance of bile salts and cholesterol cause hypercholesterolemia and elevation in serum cholesterol levels (37). Moreover, there is an increase in the activity of 3-hydroxy-3-methylglutaryl-coenzyme A (HMG-CoA) reductase following cholestasis, resulting in elevated cholesterol synthesis in the liver. During cholestasis, the expression of various transporters, which transport cholesterol from the liver into the blood, increases (38). Synthesis of apolipoprotein AI (apoA-I), the main protein component of HDL, is reduced in cholestasis and thus leads to a decrease in the serum HDL level (39). The present study showed that in BDL animals, *trans*-chalcone could increase the serum HDL level; whereas the serum levels of LDL, triglyceride, and cholesterol were reduced, which is consistent with previous studies of other animal models (7, 10, 28, 40). Prior studies have shown that *trans*-chalcone, in addition to increasing the oxidation of fatty acids, modulates the changes in lipid profile (7, 10). In HFD-induced NASH, *trans*-chalcone was effective in reducing hepatic lipogenesis by down-regulating the levels of the hepatic fatty acid synthase (FAS) enzyme, sterol regulatory element-binding protein (SREBP)-1c, SREBP-2, and peroxisome proliferator-activated receptor (PPAR)-γ2 (10). SREBP-1c, by activating lipogenic genes like FAS, stimulates lipogenesis, and SREBP-2 is involved in cholesterol biosynthesis (10, 41). PPAR-γ2 is also a potent lipogenic transcription factor. Additionally, *trans*-chalcone causes an elevation in the hepatic level of PPAR-α, which is responsible for fatty acid oxidation and inhibition of inflammation (10). Besides, in rats fed high-fat emulsion, *trans*-chalcone increases the hepatic mRNA level of sirtuin 1 (SIRT1), exerting ameliorating effects on hepatic lipid metabolism by inhibiting lipogenesis and stimulating fatty acid β-oxidation (11, 42). It has been reported that in HFD-fed rats, *trans*-chalcone administration increases the hepatic level of the ATP-binding cassette transporter A1 (ABCA1) protein, which controls lipid metabolism in the liver through HDL production or other mechanisms (11). Besides, the antifungal activity of *trans*-chalcone against *Trichophyton* rubrum is reported to be via inhibiting the synthesis of fatty acids and ergosterol (43).

The present study also showed that* trans*-chalcone could significantly reduce histopathologic abnormalities, which include inﬁltration of inﬂammatory cells, ductal hyperplasia, hepatocellular necrosis, and collagen deposition (Table 1). Hepatoprotective and antifibrotic effects of *trans*-chalcone in the CCl4-induced model of liver injury have been reported to be via reduction in the hepatic levels of collagen content, transforming growth factor-β1 (TGF-β1), and tumor necrosis factor-α (TNF-α)(8). TGF-β is involved in the activation of HSCs, leading to ECM accumulation and fibrosis (44, 45)It was also reported that trans-chalcone exerts antifibrotic effects in liver tissue through its anti-oxidant activity (7). The hepatoprotective effect of *trans*-chalcone against a high cholesterol-diet is attributed to the suppression of the angiotensin-II expression. Involvement of angiotensin-II in the induction of hepatic steatosis and fibrosis has also been previously reported (28, 46). Reduction in the mRNA level of platelet-derived growth factor (PDGF) is another mechanism by which *trans*-chalcone could decrease injury in the liver in an animal model of non-alcoholic fatty liver disease (28). It has been shown that PDGF stimulates the proliferation of HSCs and induces hepatic fibrosis (47). In HFD-fed rats, mRNA expressions of some genes associated with myocardial fibrosis, such as connective tissue growth factor, TGF-β1, and collagen type I, were also reduced by *trans*-chalcone administration (48). Anti-inflammatory properties of *trans*-chalcone in HCD-fed mice have been attributed to reduced expression of the cyclooxygenase-2 (*COX-2*) gene in hepatocyte cells (28). It has been shown that *trans*-chalcone via increasing the hepatic level of miR-451 and therefore reducing the expression of interleukin-8 (IL-8) can also decrease inflammation (11, 27). In HFD-induced pulmonary inflammation, mRNA levels of TNF-α, IL-1β, and IL-6 decreased by *trans*-chalcone administration (49).

Previous studies have reported that cholestasis induced by BDL impairs renal function, which is in agreement with our results (31, 50, 51). Our findings showed that BDL surgery caused an elevation in the serum levels of BUN and creatinine, the main indicators of renal function, and treatment with *trans*-chalcone decreased their serum levels. The renoprotective effect of *trans*-chalcone against HFD-induced kidney dysfunction has been previously reported. Alipour *et al*. indicated that *trans*-chalcone effectively protected the kidneys against HFD by elevation in the renal levels of farnesoid X receptor (FXR) in addition to reducing the levels of SREBP-1c and FAS in the kidney. FXR activation reduces the renal SREBP-1c level and thus causes a reduction in the renal triglyceride level. It was also found that *trans*-chalcone through elevation in the renal levels of FXR exerts an antifibrotic effect in the renal tissue. FXR activation down-regulates the renal levels of Smad3. Smad3 is a crucial molecule, stimulates collagen production, thereby promoting fibrosis (52).

## Conclusion

The present study demonstrated that BDL surgery significantly increased liver enzymes, BUN and creatinine, and changed lipid profile as well. Aside from observing histopathological abnormalities, BDL surgery reduced hepatic anti-oxidant activities. The trans-chalcone treatment restored the biochemical parameters to near-normal levels in a dose-dependent manner. Also, trans-chalcone revealed hepatoprotective effects by reducing liver pathological abnormalities. Considering the positive effects of trans-chalcone on liver function, it can be used to reduce cholestatic liver fibrosis complications.

## Authors’ Contributionss

M A designed the study; A A, F J, and M M performed the experiment; MA, PM, and FJ processed the data; P M analyzed and interpreted the pathological results; P MM prepared the manuscript draft; M A, P MM, and Z M edited the article; and M A supervised the experiment.

## Conflicts of Interest

None.
